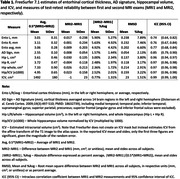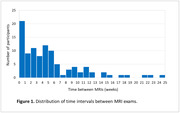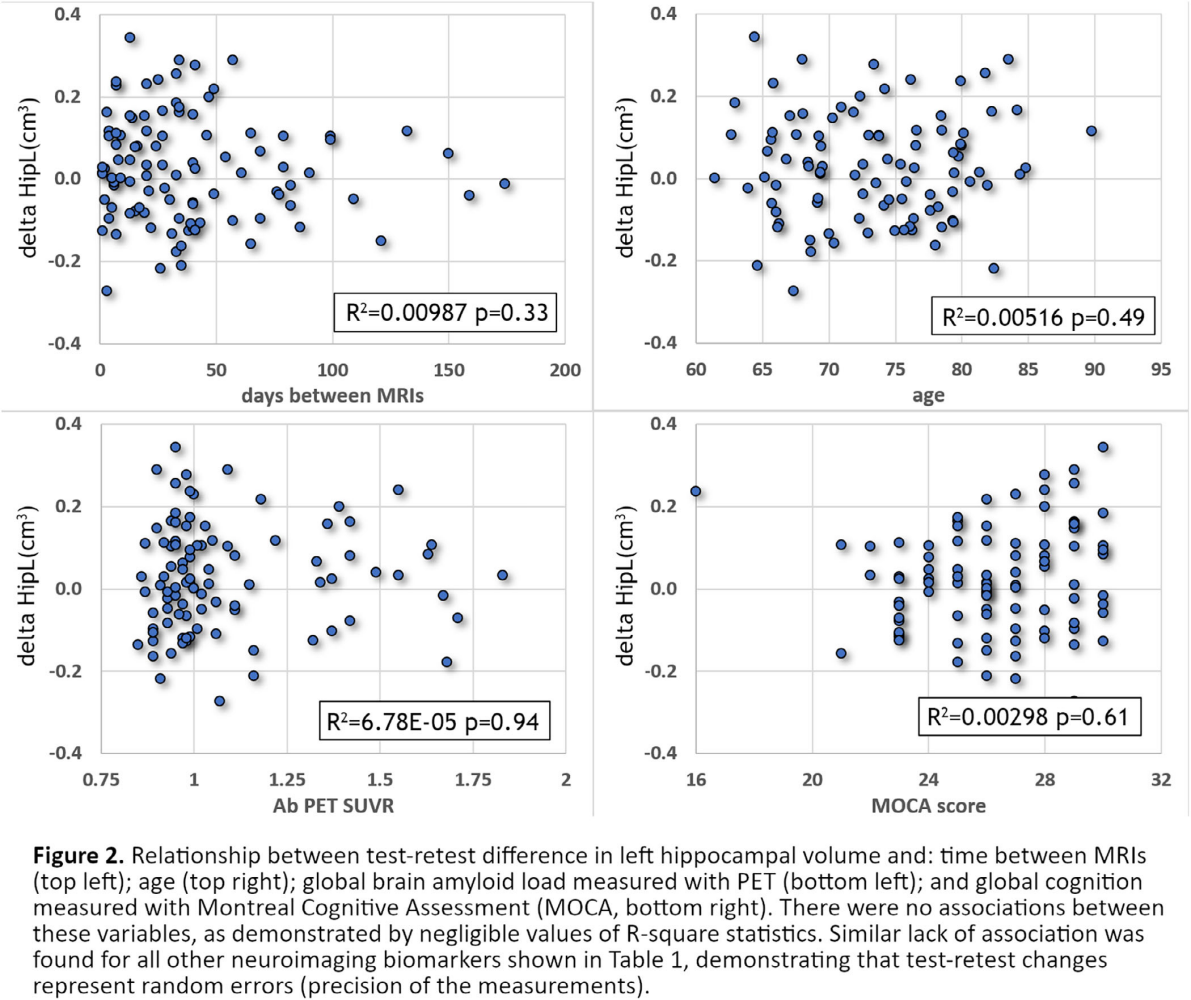# Test‐retest reliability of FreeSurfer measures of neurodegeneration

**DOI:** 10.1002/alz.085368

**Published:** 2025-01-09

**Authors:** Haiyun Chen, Louisa Bokacheva, Alok Vedvyas, Ricardo S. Osorio, Arjun V. Masurkar, Thomas Wisniewski, Henry Rusinek

**Affiliations:** ^1^ NYU Grossman School of Medicine, New York, NY USA; ^2^ NYU Alzheimer's Disease Research Center, New York, NY USA; ^3^ Alzheimer's Disease Research Center, New York University Langone Health, New York, NY USA; ^4^ Center for Sleep and Brain Health, Department of Psychiatry, NYU Langone Health, New York, NY USA; ^5^ New York University Grossman School of Medicine, New York, NY USA

## Abstract

**Background:**

FreeSurfer is widely used for monitoring neurodegeneration by quantifying MRI brain regions vulnerable to atrophic changes associated with Alzheimer’s disease (AD). FreeSurfer test‐retest reliability has been explored mainly in younger adults, but the reliability of AD imaging biomarkers is less well studied. We used two closely spaced T1‐weighted MRIs in 100 older adults to assess FreeSurfer 7.1 test‐retest reliability of AD imaging biomarkers of neurodegeneration.

**Method:**

Consecutive participants (n=100, age ≥60 years) with two brain MRI exams between Jan 2021 and Oct 2023 and less than 180 days between exams were selected from NYU Langone’s Alzheimer Disease Research Center database. Images were acquired on the same Siemens 3T PET‐MR system using 32‐channel head coil and sagittal T1‐weighted MPRAGE sequence (TR=2100ms; TE=2.98ms; TI=900ms; FA=8°; bandwidth, 260Hz/pixel; GRAPPA, 2; matrix, 256x240x176; voxel, 1x1x1 mm^3^; acquisition time, 4:35min). FreeSurfer 7.1 was used to segment the brain and determine regional volumes, cortical thickness, and intracranial volume (ICV). We compared bilateral and total hippocampal volume, entorhinal cortex thickness, AD signature (after Dickerson et al. Cereb Cortex 2009; PMID: 18632739), and ICV between two exams. Test‐retest reliability was assessed using mean absolute difference, root mean square difference, and intraclass correlation coefficient (ICC). Inter‐exam differences in AD measures were tested for association with demographic, cognitive (MOCA), and imaging features using linear regression.

**Result:**

The 100 participants (age, 73.5±6.1 years, 64% women, 33% Black, 9‐20 years education) had MRIs within an interval of 5.5±5.2 weeks (Figure 1). FreeSurfer estimates of MRI‐based biomarkers of AD were consistent between exams (Table 1). There were no associations between the differences in any of the FreeSurfer measures and inter‐MRI interval or participants’ demographic and cognitive/biological features (Figure 2). Detecting within‐individual 1% change in AD signature, whole hippocampus, or entorhinal cortex (using paired t‐test) at p=0.05 and 80% power, would require an estimated sample of 38, 69, or 347 subjects, respectively.

**Conclusion:**

Our test‐retest results reflect random errors due to image acquisition and post‐processing precision of FreeSurfer 7.1. The test‐retest reliability of most measures is excellent, except entorhinal cortex thickness. Detecting small neurodegenerative changes appears to be feasible with relatively small sample sizes.